# The evaluation of land consolidation policy in improving agricultural productivity in China

**DOI:** 10.1038/s41598-017-03026-y

**Published:** 2017-06-05

**Authors:** Xiaobin Jin, Yang Shao, Zhihong Zhang, Lynn M. Resler, James B. Campbell, Guo Chen, Yinkang Zhou

**Affiliations:** 1 School of Geographic and Oceanographic Sciences, Nanjing University, Nanjing, 210023 China; 2 Department of Geography, Virginia Polytechnic Institute and State University, Blacksburg, VA 24061 USA; 3 China Land Surveying and Planning Institute, Beijing, 10029 China; 4 Department of Geography, Michigan State University, East Lansing, MI 48824 USA

## Abstract

China is presently undergoing rapid economic development and unprecedented urbanization. Concerns over food security have prompted the Chinese government to implement large-scale land consolidation projects. However, no formal evaluation has been conducted on such projects. Thus, effectiveness of land consolidation policy remains uncertain. We obtained detailed geo-spatial information for 5328 land consolidation projects implemented between 2006 and 2010, and used time-series MODIS NDVI (2006–2016) data to assess effectiveness of China’s land consolidation policy in improving agricultural productivity. Our results show that the overall effectiveness of land consolidation in improving agricultural productivity is low, which lies in contrast to optimistic estimates based on regional statistical analysis and theoretical approaches. For projects (n = 560) implemented in 2006, about 29.5% showed significant (*p* < 0.05) increasing trends of MODIS NDVI after implementation of land consolidation. For 2007–2010, lower percentages (e.g., 25.9% in 2007 and 13.5% in 2010) of projects showed significant increasing trends. Furthermore, we found effectiveness of land consolidation projects displayed clear regional differences and driving factors are inconsistent with policy design. We anticipate our research to be a starting point for a more comprehensive evaluation involving longer time-series and higher spatial resolution data.

## Introduction

China is currently experiencing rapid economic development and unprecedented urbanization. Nationally, from 1998 to 2012, the proportion of population in urban areas has increased from 30.4% to 52.6%. In the same time period, total farmland in China reduced 7.93 million ha^1^. The challenge of using 7.63% of world’s cultivated land to feed China’s 19.78% of world’s population^2^ has attracted worldwide attention, and is one of China’s top priorities^3, 4^.

Land consolidation is regarded as an instrument or entry point for rural development^5, 6^, and an important means of improving food production capacity and reconciling land use conflict^7–9^. Land consolidation policy in China is designed mainly to mitigate farmland losses with aims to increase farmland area and improve agricultural productivity^10, 11^. At the national level, the first set of land consolidation projects in China started in 1998. During 2001–2010, 13.3 million ha farmlands have been renovated and 2.8 million ha of new farmlands were added through land consolidation projects^12^. Depending on the initial land use types and conditions, land consolidation can be grouped into three classes: farmland consolidation, land exploitation, and land reclamation. Farmland consolidation targets existing agricultural land (e.g., small parcels); land exploitation focuses on converting unused land (e.g. wild grass land, saline and alkali land, swampland, beach land, and reed land) to agricultural lands; and land reclamation aims at converting vacant/idle urban construction land and disaster-damaged farmlands back to agricultural uses. Most land consolidation practices involved amalgamation of small plots into large plots and promote construction of irrigation, drainage, roadways, and forest conservation buffers^13^. Currently, the concept of land consolidation is expanding. The Chinese government is investing more funds to implement large-scale land consolidation with goals of maintaining total farmland at the minimum of 120 million ha and increasing overall agricultural productivity by 10% by year 2020^14^.

A number of studies have evaluated the effectiveness of land consolidation projects by yield monitoring^15^, household investigation^16, 17^, statistical data analysis^18^, potential productivity estimation^19^, and agro-ecological zones analysis^20^. For example, Guo *et al*.^18^ measured the effect of land consolidation on the multifunction of farmland ecosystems for the 31 provinces of China. Using Landsat TM/ETM data from 1986 and 2000, Deng *et al*.^20^ estimated changes in land quantity and quality by agro-ecological zones analysis; Song and Pijanowski (2014)^19^ evaluated effects of farmland balance policy on potential land productivity based on national farmland natural quality grades. Findings from these studies were mixed – some showing small increases in productivity while others showing slight decreases in productivity. Until now, few studies have evaluated change of agricultural productivity of land consolidation projects at national scale, with the support of spatially explicit land consolidation parcel data. Effectiveness of land consolidation policy in improving agricultural productivity remains uncertain even though such assessment is critical for the Chinese government in improving land consolidation policy formulation and implementation.

One of the main challenges in evaluating effectiveness of land consolidation policy is obtaining parcel-level consolidation project data. We have obtained parcel boundary information of land consolidation projects (n = 5328, 2006–2010) from the “National rural land consolidation monitoring and regulation system” maintained by Ministry of Land and Resources of China. Such parcel-level data provided us the starting point to assess changes of agricultural productivity. Within each specific parcel boundary, however, in situ measures and assessment of agricultural productivity changes can be time-consuming and expensive. It is almost impossible to obtain spatially explicit agricultural productivity data (e.g., crop yield) in a retrospective manner to support long-term trend analysis. In a number of recent studies, researchers have used remote sensing-derived biophysical parameters to approximate agricultural productivity^21, 22^. For example, Yan *et al*.^22^ used satellite-derived NPP (net primary production) data from NOAA/AVHRR to evaluate agricultural productivity. The main advantage of using satellite-based NPP is that time-series data are readily available and they may serve as a common comparable unit across different crop types^23^.

Another commonly used remote sensing-derived vegetation index, the Normalized Difference Vegetation Index (NDVI), is strongly correlated with NPP and often used as a key predictor of NPP estimation^21, 24^. Time-integrated (e.g., growing season) NDVI is particularly useful as an indicator of agricultural productivity^25–28^. An increasing number of crop yield models have used time-integrated NDVI as the primary predictor for yield estimation^29–31^. Currently, standard global Moderate Resolution Imaging Spectroradiometer (MODIS) vegetation index products are provided at 250 m spatial resolution, from the early 2000s to present day. Such time-series remote sensing data provide improved capability for trend analysis (e.g., 2006–2016), a major advantage over traditional snapshots or bi-temporal analysis (e.g., 2006 vs 2016). Combining parcel boundary data for land consolidation projects and time-series MODIS NDVI data would allow us to characterize general pattern of agricultural productivity change in a site-specific way.

Additionally, few previous published studies have examined the relationship between productivity change and biophysical and socio-economic variables. For example, the biophysical characteristics of a specific land consolidation project (such as elevation, slope, shape, and area) may have positive or negative impacts on the project effectiveness. Certain climate conditions (precipitation and temperature) may promote or constrain increases in agricultural productivity. The success rate of projects may also depend on initial land use type (e.g., existing agricultural land vs vacant construction land). Statistical models could improve our understanding in project effectiveness and potentially benefit future implementation strategies of land consolidation projects. To date, studies of impacts of biophysical and socio-economic variables are rare, especially for spatially explicit settings.

In this study, a total of 5328 projects completed between 2006 and 2010 with an area greater than 100 ha were selected, for each of these projects, we assessed temporal and spatial characteristics of the effectiveness of land consolidation policy by integrating parcel-boundary data of land consolidation projects and time-series MODIS NDVI data. The main objectives of this study were to: (1) characterize the change of productivity in land consolidation parcels by analyzing time-series MODIS NDVI. We emphasize trend analysis of NDVI change, rather than bi-temporal comparison, and (2) examine association of productivity change with a variety of biophysical and social-economic variables using Generalized Linear Mixed Models (GLMMs).

## Results

### Time-series NDVI analysis

**Figure 1:**
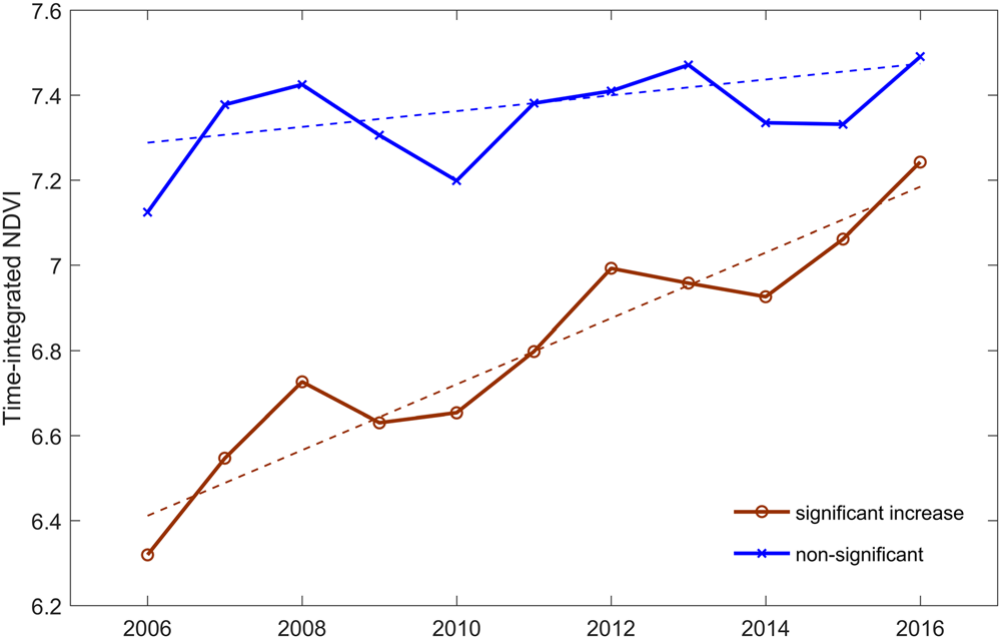
Comparison of time-integrated NDVI for two groups of 2006 land consolidation project: parcels showing significant (*p* < 0.05) upward trends as one group and all the remaining parcels as the other group. Sen’s slopes (dash lines) were estimated for two groups.

Using 250 m NDVI time-series data, we found that almost all (n = 5328) land consolidation parcels cover at least one NDVI pixel. We focused on DOY 97-DOY305 (less cloud issue) to derive time-integrated NDVI for each study year from 2006 to 2016. Figure 1 compares the averages of time-integrated (growing season) NDVI for two groups of land parcels: one group showing significant increasing trends and the other group including all remaining parcels. For parcel group showing significant increasing trends, the average increasing rate for the growing season integrated NDVI was 0.08 per year from 2006 to 2016. For projects (n = 560) implemented in 2006, a total of 165 (29.5%) projects showed significant (*p* < 0.05) increasing trends of time-integrated NDVI. For projects implemented during 2007–2010, lower percentages (e.g., 25.9% in 2007 and 13.5% in 2010) of projects showed significant increasing trends.

Table 1 compares the effective rates of three land consolidation types. Parcels with significant increasing trends in NDVI were considered as effective and the remaining parcels were considered as non-effective. When all projects from 2006 to 2010 were included for comparison, Land Reclamation (LR) projects appeared to have the highest effective rate (28.3%) while Farmland Consolidation (FC) and Land Exploitation (LE) projects showed relatively lower effective rates of 17.3% and 23.9%. Such results were expected because LR projects aimed at converting vacant/idle urban construction land and disaster-damaged farmland back to agricultural uses. The initial NDVI values for LR projects thus were relatively low and continuous increases of NDVI was expected. We note that project effectiveness varied substantially across time, projects implemented earlier showed higher effective rates. Such a result is expected because it may take a few years to show the project effectiveness, especially observed from remote sensing data. For projects implemented in 2010, only 7 years of NDVI data (2010–2016) were included for analysis. Interpretation of trend analysis using 7 years of data need to apply with caution.

**Table 1:** The proportion of land consolidation project achieving effectiveness.

	Farmland Consolidation	Land Exploitation	Land Reclamation	All projects
2006	29.6 (412)	29.3 (133)	26.7 (15)	29.5 (560)
2007	23.9 (635)	36.7 (98)	34.8 (23)	25.9 (756)
2008	18.5 (610)	35.9 (153)	29.6 (27)	22.3 (790)
2009	14.8 (695)	28.7 (129)	31.8 (22)	17.4 (846)
2010	12.8 (1810)	15.7 (540)	19.2 (26)	13.5 (2376)
2006–2010	17.3 (4162)	23.9 (1053)	28.3 (113)	

Note: The ratio is computed by dividing the number of effective parcel by total parcel number. The number inside the brace represents the total parcel of certain type.

**Figure 2:**
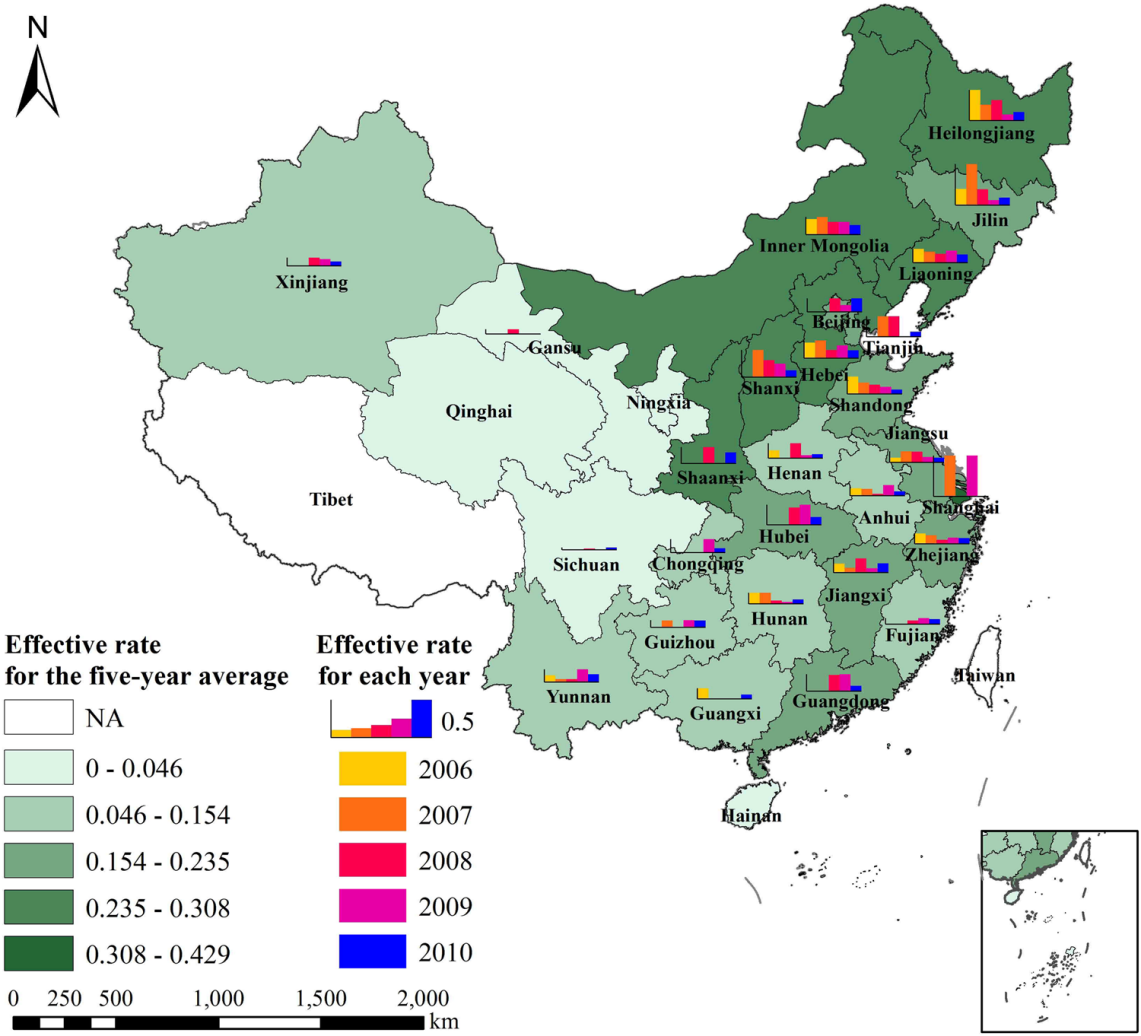
The effective rates (i.e., effective projects/total number of projects) was calculated for each province in China. Map was generated using ArcGIS 10.3 (http://www.esri.com/).

We further evaluated the effective rate at province-level (Fig. 2). Overall, the effective rate was low for the east, south, and southwestern China. Note that the east and south China have the most productive lands. Agricultural productivity was probably already maximized before new projects were established. Southwest China has relatively complex topographical conditions, poor farmland quality, and limited water conservancy infrastructure that present a challenge for project implementation. The north, central and northwestern regions had high effective ratio. In these regions, farmland quality is dominated by good-to-medium levels and a single, irrigated, crop system is often used. Projects in these regions showed promising results as observed by from remote sensing.

### Association between NDVI trends and biophysical and social-economic variables

**Figure 3:**
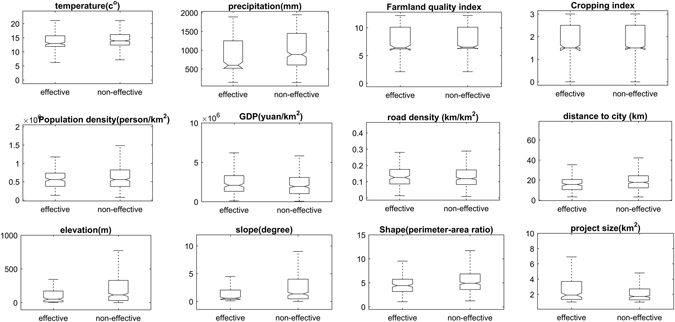
Comparison of selected biophysical and social-economic variables for effective and non-effective sites. Parcels with significant increasing trends in NDVI were considered as effective and the remaining parcels were considered as non-effective.

Figure 3 compares selected biophysical and social-economic variables for effective and non-effective sites of 2006 projects. Effective sites were associated with lower temperature, lower precipitation, lower elevation, and smaller slope. For socio-economic variables, effective sites located in areas relatively closer to city, higher GDP, and lower farmland quality index (using National Farmland Quality Grades as farmland quality index, a lower value represents a higher average potential productivity. More details are available in Supplementary Table S1). Overall, there were less clear separations for socio-economic variables compared to biophysical variables.

Table 2 shows GLMM analysis results. We hypothesized that there were variations of the intercept across provinces. The variance should be 0 or close to 0 if there was no between-province variability. The variance for the GLMM model was 0.07 and an Exact (Restricted) Likelihood Ratio Tests for Mixed and Additive Models (RLRsim) testing showed the variance was significantly (*p* < 0.01) different from 0, suggesting the importance of keeping the random effects in the model.

**Table 2:** GLMM model results showing the relationship between project effectiveness and a variety of biophysical and socio-economic variables.

	Coefficients	*p*
Fixed effects		
(Intercept)	1.03	0.102
temperature	−0.739	0.625
precipitation	−3.427	**0**.006**
farmland quality index	1.996	0.269
population density	−0.886	0.212
GDP	0.625	0.501
road density	−2.262	**0**.003**
distance to city	−1.562	**0**.070*
elevation	−1.357	**0**.050**
slope	−1.911	**0**.059*
shape	1.132	0.343
area	2.034	**0**.037**
factor (land exploitation)	−0.180	0.483
factor (and land reclamation)	−0.418	0.520
Random effects	Variance	Std.Dev.
Province (Intercept)	0.07	0.25

The dependent variable is binary (code 1 = projects showing significant increasing NDVI trends, code 0 = all the remaining projects).

GLMM showed that project effectiveness was negatively (*p* < 0.05) associated with precipitation, road density, and elevation. For example, an increase in precipitation is associated with decrease in the expected log odds of project effectiveness. Controlling for similarities in other biophysical and socio-economic variables, projects in south and east China were less likely to be effective considering the higher average annual precipitation in that region. GLMM results also indicate that projects located in more developed areas (e.g., higher road density) were also less likely to be effective. A possible reason for this finding is that developed regions are clustered in the east and south China, where agricultural productivity might already be maximized. In addition, projects effectiveness was negatively associated with slope and distance to city at 0.1 significance level.

Project effectiveness was positively (*p* < 0.05) associated with project size. Impacts of project size was also expected – a project with larger size was more likely to be labelled as effective. Among three land consolidation project types, land exploitation and land reclamation had lower odds of success (non-significant) compared to the reference class of farm consolidation. Such results were valid for 2006 projects only, because we observed higher effective rates for land exploitation and land reclamation projects in other project years (Table 1). Variables showed non-significant association include annual temperature, farmland quality index, population density, GDP, and shape index. It was surprising to see that site-specific characterizes such as farmland quality index and shape index were not important in the model. These data may have coarse spatial resolution and rather broad definition of farmland quality; thus they may not be useful in linking project effectiveness.

## Discussion

China’s land consolidation policy aims to improve overall agriculture productivity through expansion of farmland, improving agriculture infrastructure, increasing irrigated cropland, and improving farmland use efficiency. According to ‘National land consolidation planning (2011–2015)’, 26.7 million ha of well-developed farmland (22% of total national farmland) will be established through land consolidation programs by 2015 (this goal was achieved at the end of 2015)^32^ and a further 26.7 million ha will be completed by 2020 to lay the foundation for improving quality of farmland. Therefore, assessing effectiveness of land consolidation policy in agricultural productivity is critical for the Chinese government to improve land consolidation policy formulation and implementation to guarantee national food security.

We conducted a national scale evaluation of project effectiveness for a total of 5328 projects using time-series analysis of MODIS NDVI (2006–2016) products. For projects implemented in 2006, we were expecting that most parcels would show significant increasing trends in time-integrated NDVI, because such projects were implemented earlier and impacts should be observable from time-series remote sensing data. However, only 29.5% parcels showed significant increasing trends after project implementation. Similar or lower effective rates were observed for projects implemented from 2007 to 2010.

**Figure 4:**
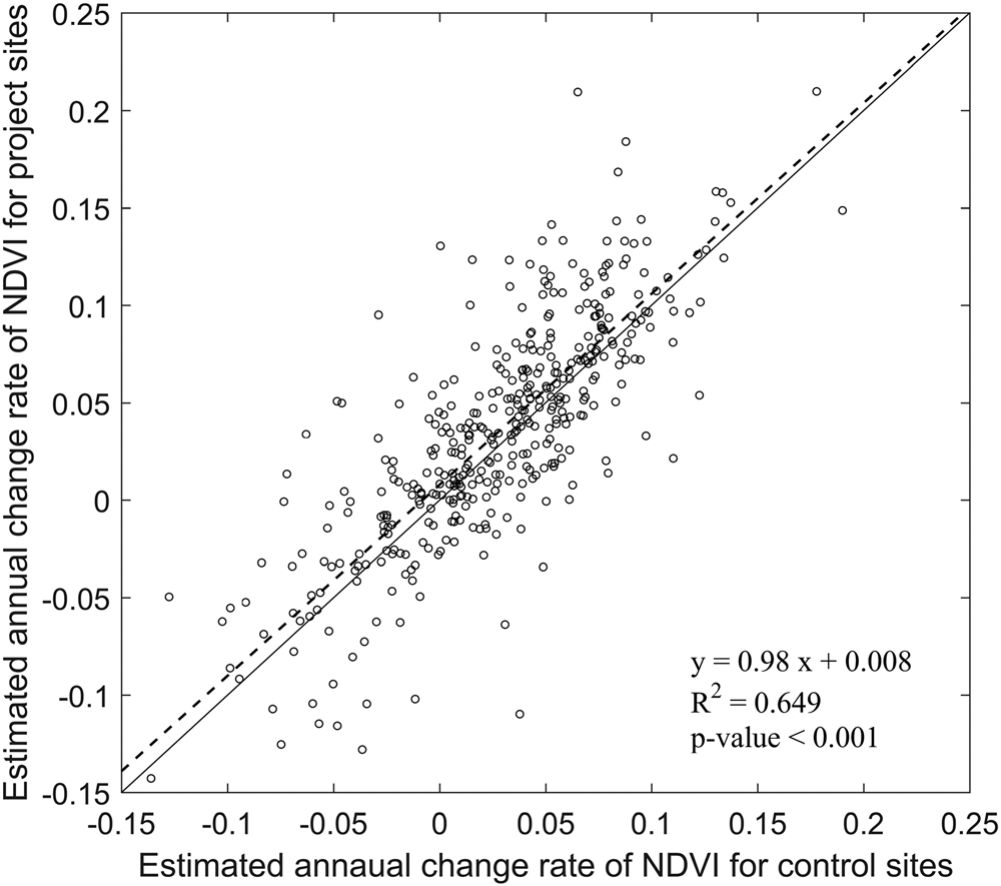
Comparison of NDVI change rates (i.e., Sen’s slope for 2006–2016) of project sites and their corresponding control sites. The 2006 farmland consolidation projects (n = 412) were used for pair comparison. Dash line denotes the regression line.

We note that the effective rate can vary substantially depending on how researchers design project evaluation method. For our study, additional analyses were conducted for 2006 projects (farmland consolidation projects only, n = 412) by introducing control sites for pair comparison. Specifically, for each project site, an arbitrary 3-km buffer was created and we extracted the project’s immediate surrounding agricultural area as a control site for evaluating NDVI change rates. Sen’s slope estimator was used to calculate NDVI change rates from 2006 to 2016. Figure 4 compares NDVI change rates of project sites and their corresponding control sites. For each point, y-axis represents estimated NDVI change rate for the project and x-axis represents estimated change rate for the corresponding control site. About 60.4% of projects show higher Sen’s slope values (or positive impacts) compared to control sites. With such a simple comparison, a higher percentage of projects may be considered as effective. However, it is unclear whether a project is truly effective if its NDVI change rate was only marginally higher than the corresponding control site.

As for regional differences, effective projects concentrated in the North China Plain and the Yellow River Delta. Through an analysis of driving factors, effective projects are more likely to locate in areas with lower annual average temperature and precipitation, and a lower level of economic development. However, there are still a significant number of land consolidation projects in areas with initially poor land quality that did not achieve noticeable improvement.

The effectiveness of land consolidation policy in improving agricultural productivity does not appear promising based on remote sensing observation. Several possible explanations include disruption of natural resources, discrepancy of management capabilities, and arbitrary site selection. Generally, ineffective land consolidation may arise from four sources. First, it may be caused by contradictions between the central government and local governments in initiating land consolidation policies. Specifically, the central government emphasizes food security, environment and resources protection while local governments mainly target at economic development. Secondly, over-emphasizing the standard of plots into a rectangle, trees in rows, roads interlinked, and canals adjacent and equipped to irrigate and drain may actually lead to undesirable agricultural productivity outcomes. Bevis & Barrett (2016)^33^ described an inverse-productivity relationship – smaller farms can be more productive compared to the larger ones. Such findings and related theories need to be considered in the future land consolidation policy design. Thirdly, the lack of efficient connections between land consolidation policy and other social-economic plans (such as urban expansion plans, rural development plans and environment protection plans) may induce lower efficiency of land use. The lack of long-term investment and continuous policy support directly leads to difficulties in landownership adjustment or transfer.

For future improvement of land consolidation policy, we suggest:

- in addition to the “top-down” method of policy implementation, the “bottom-up” operation strategy should also be incorporated. Contemporarily in China, the national plan is a “one-size-fits-all” solution for all local plans. The impacts of land consolidation might be weakened by the discrepancies across different administrative levels. A “bottom-up” operational strategy should be incorporated and combined with the traditional “top-down” land consolidation administrative style to harbor differences in regional land use structures and local socio-economic contexts.
- besides the requirement of an annual goal of total land consolidation projects and areas, agriculture productivity evaluation should be routinely conducted. Until now, dynamic monitoring and evaluation mechanism are entirely missing with regard to land consolidation progress. In this context, local governments generally neglect comprehensive assessments of local farmland resources, resulting in the randomness in locating land consolidation projects. Accordingly, Chinese central government cannot acquire a comprehensive picture regarding the effectiveness of land consolidation programs and the challenges faced.

Our research highlights the use of time-series remote sensing data to assess the effectiveness of land consolidation projects. Although there are concerns of using such moderate resolution (250 m) data (e.g., pixel purity and pixel-target adequacy problems)^34, 35^, a significant portion of projects did not show increasing trends, which may not be explained by spatial resolution and geometric uncertainty alone. We also note that only up to 11 years (2006–2016) of remote sensing data were used for the trend analysis. Projects involved in this study were mainly implemented during 2006–2010. Some projects may need longer time-series data to assess the actual impacts. Overall, our research serves as a starting point for more comprehensive evaluation which may involve longer time-series and higher spatial resolution remote sensing data, field validation, and various statistical analysis.

## Data and Methods

### Data

**Figure 5:**
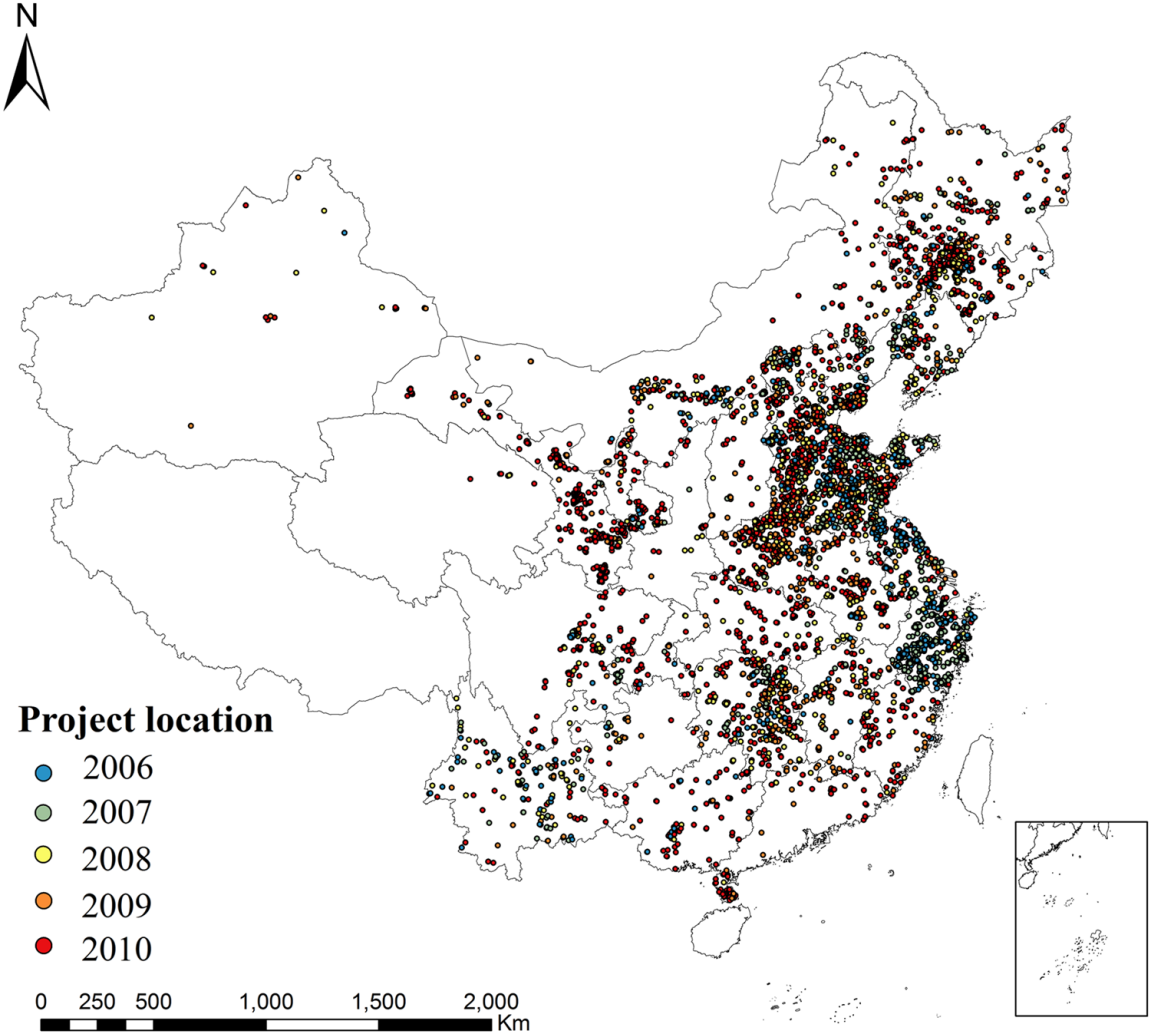
Location of selected land consolidation projects. Map was generated using ArcGIS 10.3 (http://www.esri.com/). Notes: The average size of selected land consolidation parcel was 5.56 km^2^ and the largest project covered an area of 69.64 km^2^. Among the selected 5328 projects, the earliest projects (n = 560) completed in 2006 and the latest projects (n = 2376) completed in 2010. By provinces in China, Shandong completed a highest number of 824 projects (total area = 3549.49 km^2^), and Tibet and Hainan did not complete any project during 2006 to 2010. On average, land consolidation projects in Jilin and Guizhou had the largest (11.86 km^2^) and smallest (1.92 km^2^) mean parcel size, respectively. The selected land consolidation projects can be further grouped to three types described previously: a total of 4162 projects focused on farmland consolidation, the average parcel size was 5.80 km^2^; another 1053 projects focused on land exploitation with average parcel size of 4.88 km^2^; the remaining 113 projects focused on land reclamation and the average parcel size was 3.15 km^2^.

A total of 5328 projects with size greater than 100 ha were selected for this research (Fig. 5). Detailed information of projects, including center location, land parcel boundary, parcel area and construction completion date, were obtained from the “National rural land consolidation monitoring and regulation system” maintained by Ministry of Land and Resources of China.

The 250 m Terra-MODIS 16-day composites of NDVI data (MOD13Q1, collection 5) from 2006 to 2016 were downloaded from the NASA Reverb website (http://reverb.echo.nasa.gov/). For each compositing window (e.g., DOY 1, 2006), 17 NDVI scenes for the study area were mosaiced and then re-projected to Albers Equal Area Conic projection using MODIS Reprojection Tool (MRT). For the entire study period of 2006–2016, a total of 4301 MODIS NDVI scenes were processed to provide time-series data for entire China. All compositing image mosaics were then stacked to form 250 m time-series NDVI image cube for China, 2006–2016.

A variety of GIS data layers were developed to assess their impacts on agricultural productivity in land consolidation projects. Specifically, we developed twelve explanatory variables, which can be classified into four categories: (1) climate variables including annual temperature and annual precipitation; (2) land use related variables including farmland quality index and cropping system; (3) social-economic variables including population density, GDP, road network density, and distance to city; (4) project characteristics variables including elevation, slope, shape index and project size. Detail of these data, including definition, source, resolution, and description are provided in Supplementary Table S2.

## Methods

### MODIS NDVI analysis

The spatial boundaries of land consolidation projects were used to extract MODIS NDVI values. We note that small land parcels (e.g., <100 ha) were not included in this study since they cover limited number of MODIS NDVI pixels. Each year’s MODIS NDVI product has 23 composite layers, thus for each year we extracted 23 NDVI values to build an annual time series of 16-day NDVI composite values. We further evaluated the Reliability Index (RI) of MODIS NDVI pixels and found that a large number of MODIS NDVI pixels had low reliability (RI > 1) during winter seasons (November-March) due to snow cover. Therefore, we focused on mid-April to early-November (DOY 97-DOY 305) for each year to calculate time-integrated NDVI value. Within each land parcel, time-integrated NDVI values were average for all contributing MODIS pixels to represent parcel-level NDVI integral. The final product for the NDVI data was a 5328 by 11 matrix, representing change of overall greenness for 5328 projects, 2006–2016.

For each land parcel, we evaluated NDVI trends after each project’s implementation time. For example, for projects implemented in 2006, we evaluated NDVI trends from 2006 to 2016. For projects implemented in 2007, we evaluated NDVI trends from 2007 to 2016. We used the Mann-Kendall test to characterize the temporal trend of yearly NDVI. The Mann-Kendall test is a nonparametric method commonly used to test and model temporal trends. One appealing feature of the Mann-Kendall test is its robustness to outliers^36^. The null hypothesis (H_0_) is that there is no monotonic (upward or downward) trend in the time series data, while the alternative hypothesis (Ha) suggests that monotonic trend is present. For land parcels showing significant (*p* < 0.05) upward or downward trends, we estimated the average annual change rate using Sen’s slope estimator. Land parcels showing significant upward trends in NDVI were considered as effective projects and all the remaining projects were considered as non-effective from a remote sensing data analysis perspective. For projects implemented in 2006, we further compared projects’ NDVI trends with those from their immediate surrounding (i.e., within 3 km buffer) agricultural area. The Sen’s slopes for project sites and their paired control sites were compared to evaluate project effectiveness. Finally, the spatial distribution of effective rates (i.e., effective projects/total number of projects) was calculated for each province in China and illustrated in a GIS map to assess spatial/regional differences.

### Association between project effectiveness and biophysical and socio-economic variables

A number of statistical methods can be used to understand the associations between project effectiveness and selected biophysical and social-economic variables. For each selected variable, we first used box plots to visualize the differences between effective and non-effective sites. The effective sites were defined as those parcels showing significant upward trends of NDVI and the non-effective sites including all the remaining sites.

Considering that the land consolidation projects were located in 29 provincial-level administrative units and project implementation practices may vary across provinces, we hypothesized a province-level random effect on project effectiveness. A Generalized Linear Mixed Model (GLMM) was used to predict project effectiveness incorporating selected biophysical, social-economic variables as fixed effects and province variables to model random effects. Such hierarchical model has advantages in quantifying the variation among study units (e.g., provinces)^26, 37^. Equation (1) is a general equation of the GLMM:

1 $${\rm{g}}({\mu })=\,X\beta +Zb+\,\delta $$

where *µ* is the expected effectiveness of land consolidation project, *X* is the predictor matrix including all biophysical and social variables described in the data section. *β* is a vector of regression coefficients for these fixed factors. *Z* is the random-effects design matrix (e.g, province-level) and *b* is regression coefficients for random-effects. *δ* represents model residuals. We used the lme4 package in R program to develop the GLMM^38^. For response variable, land parcels showing significant upward trends in NDVI were coded as 1 (effective sites) and all other parcels were coded as 0. Additional independent variable such as project type (e.g., farmland consolidation, land exploitation, and land reclamation) was also considered in model development. We examined multicollinearity among independent variables using Pairwise Pearson correlation analysis. The highest Pearson correlation value was less than 0.2, thus all independent variables were included in the model. Akaike’s Information criterion (AIC) was used to select the best fit model.

## Electronic supplementary material

Supplementary tables
