# Supplementary material for: The evaluation of land consolidation policy in improving agricultural productivity in China
Source: Sci Rep. 2017 Jun 5;7:2792. doi: 10.1038/s41598-017-03026-y (PMC5459834; doi:10.1038/s41598-017-03026-y)
Supplement: Supplementary file 1 — Supplementary tables [file 41598_2017_3026_MOESM1_ESM.pdf]

Supplementary Material for

The evaluation of land consolidation policy in improving agricultural productivity in China

Xiaobin Jin [a], Yang Shao [b], Zhihong Zhang [a,c], Lynn M. Resler [b], James B. Campbell [b], Guo Chen[d], Yinkang Zhou [a]

a) School of Geographic and Oceanographic Sciences, Nanjing University, Nanjing 210023, China; b) Department of Geography, Virginia Polytechnic Institute and State University, Blacksburg, VA 24061, USA; c) China Land Surveying and Planning Institute, Beijing 10029, China; d) Department of Geography, Michigan State University, East Lansing, MI 48824.

List of contents:

Table S1 Area of land consolidation of study projects from 2006 to 2010

Table S2 Data description of explanatory variables

Table S1 Area of land consolidation of study projects from 2006 to 2010 (km^2)

Province	2006	2007	2008	2009	2010	2006-2010
Anhui	23.12	13.74	36.17	47.57	105.67	226.28
Beijing	0.00	8.88	9.23	13.74	10.21	42.06
Fujian	5.25	0.00	21.18	24.17	40.95	91.55
Gansu	3.70	23.94	32.42	42.93	514.90	617.88
Guangdong	0.00	0.00	9.34	35.35	71.21	115.90
Guangxi	32.91	0.00	17.54	15.79	68.90	135.13
Guizhou	2.72	8.64	3.12	19.80	10.74	45.02
Hainan	0.00	9.79	9.20	1.26	7.67	27.92
Hebei	173.15	138.85	297.83	247.78	329.45	1187.06
Henan	6.08	6.42	138.67	605.07	815.30	1571.55
Heilongjiang	6.19	67.20	10.29	174.02	257.95	515.65
Hubei	0.00	1.54	44.90	1.57	160.71	208.72
Hunan	50.49	68.26	161.94	193.77	159.40	633.86
Jilin	74.83	1.04	155.68	199.75	987.55	1418.84
Jiangsu	148.96	184.19	147.49	130.19	131.96	742.80
Jiangxi	21.51	31.79	84.37	167.80	128.62	434.09
Liaoning	29.61	122.51	129.93	70.55	102.85	455.44
Inner Mongolia	71.33	49.89	101.15	143.39	168.25	534.02
Ningxia	0.00	0.00	8.41	34.28	123.17	165.86
Qinghai	0.00	0.00	27.01	0.00	12.46	39.48
Shandong	384.16	503.25	567.43	431.48	482.46	2368.78
Shaanxi	9.81	11.01	35.60	79.74	14.37	150.53
Shanxi	0.00	1.79	18.49	13.12	19.31	52.71
Shanghai	0.00	3.14	0.00	3.64	4.01	10.80
Sichuan	21.93	38.63	168.82	34.02	184.71	448.11
Tianjin	0.00	0.00	2.74	3.03	1.51	7.28
Xinjiang	2.00	2.00	12.51	20.40	38.69	75.59
Yunnan	79.43	58.20	97.72	48.71	62.07	346.12
Zhejiang	285.20	519.28	62.98	35.29	16.03	918.79
Chongqing	3.07	31.94	8.10	9.29	46.96	99.34
**Total**	**1435.45**	**1905.90**	**2420.26**	**2847.49**	**5078.03**	**13687.13**

Table S2 Data description of explanatory variables

Data	Unit	Resolution	Source	Description	Detail
Land consolidation project			The National Rural Land Consolidation Monitoring and Regulation System (RLCMRS) maintained by Ministry of Land and Resources of China	Including statistics of accepted land consolidation projects at all administration levels	Including spatial boundary and project records i.e. location, area, newly added farmland, investment, and construction completion date.
Land use		1:250 000	Earth-system Scientific Data Sharing Platform		Based on remote sensing image and ground information of 2005
Temperature	℃	1km*1km	National Meteorological Information Center (http://http://www.nmic.gov.cn)	The average annual temperature during 2001-2010	Related data are recorded by meteological stations of China Meteological Administration, which were interpolated into 1km resolution grid data with the Kriging interpolation algorithm.
Precipitation	mm	1km*1km		The average annual precipitation during 2001 -2010	
Elevation	m	90m*90m	Resources and Environment Science Data Center, Chinese Academy of Sciences (http://www.resdc.cn)	The average elevation of grid	
Slope	°	90m*90m		The average slope of grid	
Farmland quality index	Grade	1:500 000	National Farmland Quality Grades	According to the potential land productivity values of farmland ranked categories numbered 1-15. With smaller grade representing higher average potential productivity and each grade represents 1500 kg/ha potential productivity. The nationwide data	Derived from assessments of photosynthetic-thermal-water-land potentiality, it is similar to the method that the United Nations Food and Agriculture Organization and the International Institute for Applied Systems Analysis employed to identify Agro-Ecological Zones.

				collection was finished in 2008.	
Multiple cropping index		1:4 000 000	National Science and Technology Infrastructure (http://region.agridata.cn)	Based on the crop type and planting system, the Chinese mainland was partitioned to 13 farming zones.	Valuing 1 for one cropping crops, 1.5 for double cropping or one cropping, 2 for double cropping, 2.5 for double cropping and early tri cropping, 3 for tri cropping.
Population	10^4/km^2	county	Data Sharing Infrasture of Earth System Science (http://www.geodata.cn)	The county average population in 2010.	
GDP	10^4RMB/km^2	county		The county average GDP (Gross Domestic Product) in 2010.	
Road network density	km/km^2	1km*1km	The Beijing City Lab (http://www.beijingcitylab.com)	Length of high-level road per unit area in 2010	High-level road including highway, state roads and provincial roads
Distance to city	km	polygon	RLCMRS	The distance between the center of project to the center of the nearest city	
Shape index		polygon	RLCMRS	Perimeter-to-area ratio for a parcel	
Project size	km^2	polygon	RLCMRS	The area of a parcel	
